# A case report of myocarditis combined with hepatitis caused by herpes simplex virus

**DOI:** 10.1186/s12872-018-0869-2

**Published:** 2018-07-03

**Authors:** Tetsuya Yamamoto, Tsuneaki Kenzaka, Masanori Matsumoto, Ryo Nishio, Satoru Kawasaki, Hozuka Akita

**Affiliations:** 1 Department of Internal Medicine, Hyogo Prefectural Kaibara Hospital, 5208-1, Kaibara, Kaibara-cho, Tanba, Hyogo, 669-3395 Japan; 2 Division of Community Medicine and Career Development, Kobe University Graduate School of Medicine, 2-1-5, Arata-cho, Hyogo-ku, Kobe, Hyogo 652-0032 Japan

**Keywords:** Myocarditis, Hepatitis, Herpes simplex virus, Case report

## Abstract

**Background:**

Viral myocarditis presents with various symptoms, including fatal arrhythmia and cardiogenic shock, and may develop into chronic myocarditis and dilated cardiomyopathy in some patients. We report a case of viral myocarditis and hepatitis caused by herpes simplex virus.

**Case presentation:**

A 20-year-old woman was admitted to our hospital with fever, fatigue, and anorexia. The initial investigation showed elevated liver enzyme levels and elevated creatine phosphokinase, and computed tomography showed diffuse swelling and internal heterogeneous image in the liver. These findings were consistent with acute hepatitis; therefore, we performed a liver biopsy, which showed parenchymal necrosis and lymphocytic infiltration. The night that the liver biopsy was performed, blood pressure gradually decreased and revealed cardiogenic shock. Electrocardiography showed diffuse ST-segment elevation, and echocardiography showed a dilated, spherical ventricle with reduced systolic function and pericardial effusion. An endomyocardial biopsy revealed lymphocyte infiltration of the myocardium, confirming acute myocarditis. After a few days, tests for immunoglobin M and immunoglobin G antibodies against herpes simplex virus were positive.

**Conclusions:**

We presented a rare case of myocarditis combined with hepatitis that was caused by herpes simplex virus. Acute myocarditis can occur concurrently with hepatitis, pancreatitis, nephritis, and encephalitis; thus, determining the presence of other infectious lesions is necessary to provide appropriate treatment for the patient.

## Background

Myocarditis can present with various symptoms, ranging from mild dyspnea to chest pain, cardiogenic shock, and fatal arrhythmia. The main cause of myocarditis is current or recent viral infection [1]. Enteroviruses, specifically Coxsackievirus (CV) group B serotypes, have traditionally been perceived as the predominant viral cause [2], although adenoviruses, parvovirus B19, and human herpes virus 6 can also cause myocarditis [3–5]. In contrast, herpes simplex virus (HSV) rarely causes acute myocarditis. A few case reports described that viral myocarditis may be combined with hepatitis, pancreatitis, nephritis, and encephalitis [6–8].

We encountered a case of combined myocarditis and hepatitis caused by HSV infection.

## Case presentation

A 20-year-old woman, who had an unremarkable medical history and was immunocompetent, was admitted to another hospital due to fever, fatigue, and anorexia, and she was administered acetaminophen and antibiotics. She also experienced vomiting, as well as systemic myalgia 5 days after admission causing an inability to move. Her condition was worsening, and she was transferred to our hospital 7 days after her initial admission. Upon admission, her liver enzyme and creatine phosphokinase (CPK or CK) levels were high. She had no history of jaundice, pruritus, clay stools, melena, hematemesis, abdominal distension, or altered sensorium. She reported only an occasional small amount of ethanol intake and had not had sexual intercourse. The patient denied intake of indigenous medicine or intoxication. The patient did not report any past major surgeries, blood transfusions, or intravenously injected drug abuse prior to onset of the disease. Additionally, she did not report any history of diabetes, hypertension, tuberculosis, thyroid disease, trauma, exposure to industrial toxins or radiation, blood or blood component therapy, bleeding disorders, promiscuity, or similar complaints in the family or neighborhood.

Upon admission, her vital signs were as follows: body temperature, 37.2 °C; blood pressure, 110/72 mmHg; pulse, 75 beats/min; respiratory rate, 20 breaths/min; and oxygen saturation, 98% on room air. A physical examination revealed mild enlargement of the liver, no pitting edema in both legs, and no coarse crackles over the lung fields. Laboratory findings are presented in Table 1.

**Table 1:** Laboratory data upon admission

Parameter	Recorded value	Standard value
White blood cell count	10,060/μL	4500–7500/μL
Neutrophils	56.9%	
Hemoglobin	16.4 g/dL	11.3–15.2 g/dL
Hematocrit	47.6%	36–45%
Platelet count	12.6 × 10^4^/μL	13–35 × 10^3^/μL
International normalized ratio	1.19	0.80–1.20
Activated partial thromboplastin time	33.4 s	26.9–38.1 s
C-reactive protein	1.52 mg/L	≤1.0 mg/L
Total protein	6.8 g/dL	6.9–8.4 g/dL
Albumin	4.0 g/dL	3.9–5.1 g/dL
Total bilirubin	0.8 mg/dL	0.2–1.2 mg/dL
Aspartate aminotransferase	2082 U/L	11–30 U/L
Alanine aminotransferase	1824 U/L	4–30 U/L
LDH	4191 U/L	109–216 U/L
LDH-1	12.9%	
LDH-2	15.2%	
LDH-3	13.3%	
LDH-4	18.2%	
LDH-5	38.7%	
CK	3753 U/L	40–150 U/L
CK-MM	99.4%	
CK-MB	0.6%	
Blood urea nitrogen	38.8 mg/dL	8–20 mg/dL
Creatinine	0.8 mg/dL	0.63–1.03 mg/dL
Sodium	134 mEq/L	136–148 mEq/L
Potassium	5.2 mEq/L	3.6–5.0 mEq/L
Chloride	106 mEq/L	98–108 mEq/L
Glucose	108 mg/dL	70–109 mg/dL
pH	7.474	7.350–7.450
Partial pressure of carbon dioxide	34.9 mmHg	35.0–45.0 mmHg
Bicarbonate ion	25.1 mEq/L	23.0–28.0 mEq/L
Lactic acid	3.04 mmol/L	0.44–1.78 mmol/L
Anion gap	11.9 mEq/L	10.0–14.0 mEq/L

*LDH* lactate dehydrogenase, *CK* creatine phosphokinase, *CK-MM* CK in the skeletal muscle, *CK-MB* CK in the blood

**Fig. 1:**
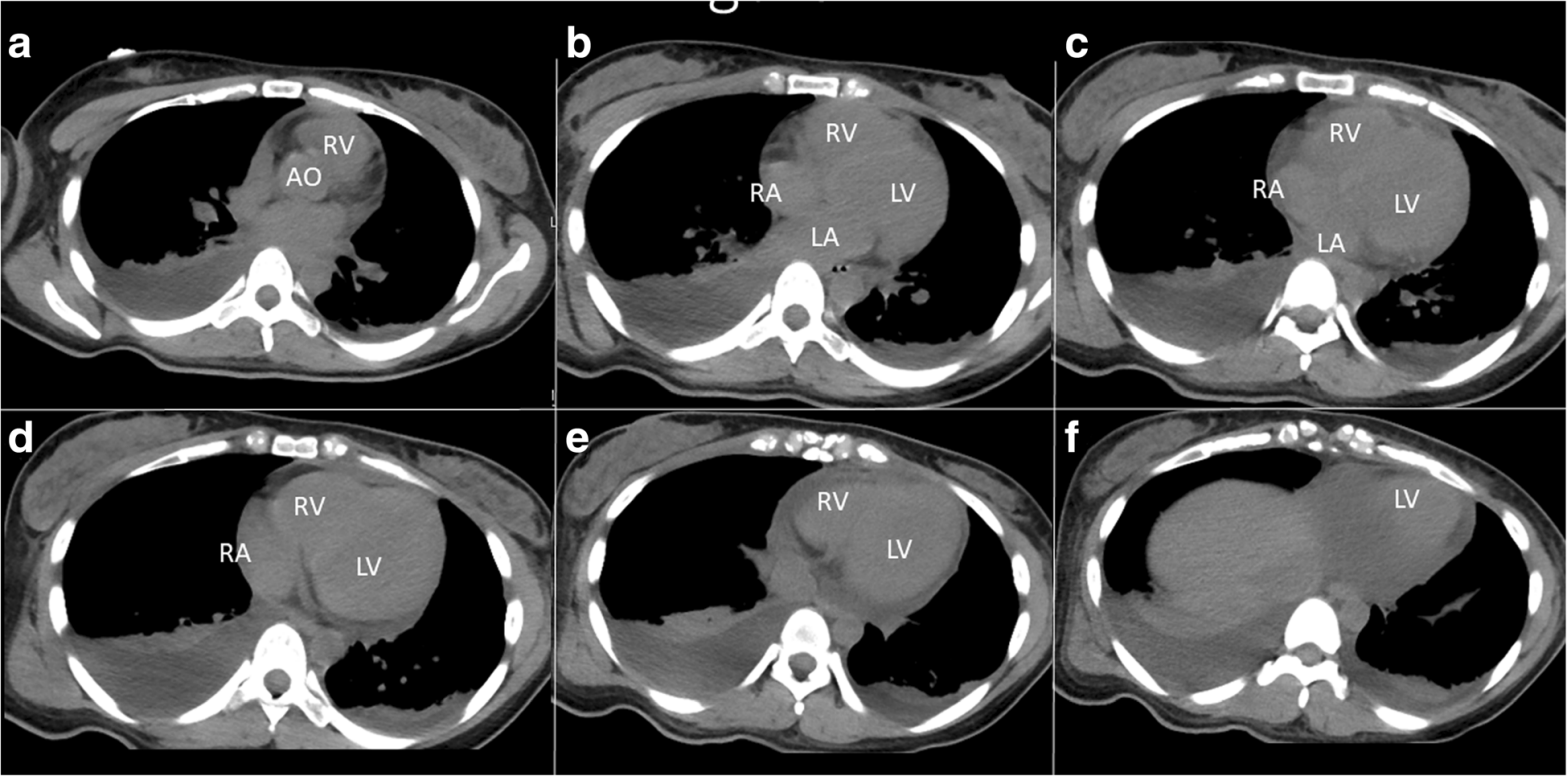
Computed tomography of the heart. (**a-f**) Consecutive slices in horizontal view. Scans revealed a minimal pericardial effusion. Ao, ascending aorta; RA, right atrium; RV, right ventricle; LA, left atrium; LV, left ventricle

Additionally, a chest radiography showed absence of pulmonary congestion, pleural effusion, and cardiomegaly. Electrocardiography was not performed on admission. Computed tomography of the chest (Fig. 1) and abdomen revealed minimal pericardial effusion, diffuse swelling, and an internal heterogeneous image in the liver. These findings were compatible with acute hepatitis; therefore, we did not examine for cardiac function despite the presentation of pericardial effusion.

**Fig. 2:**
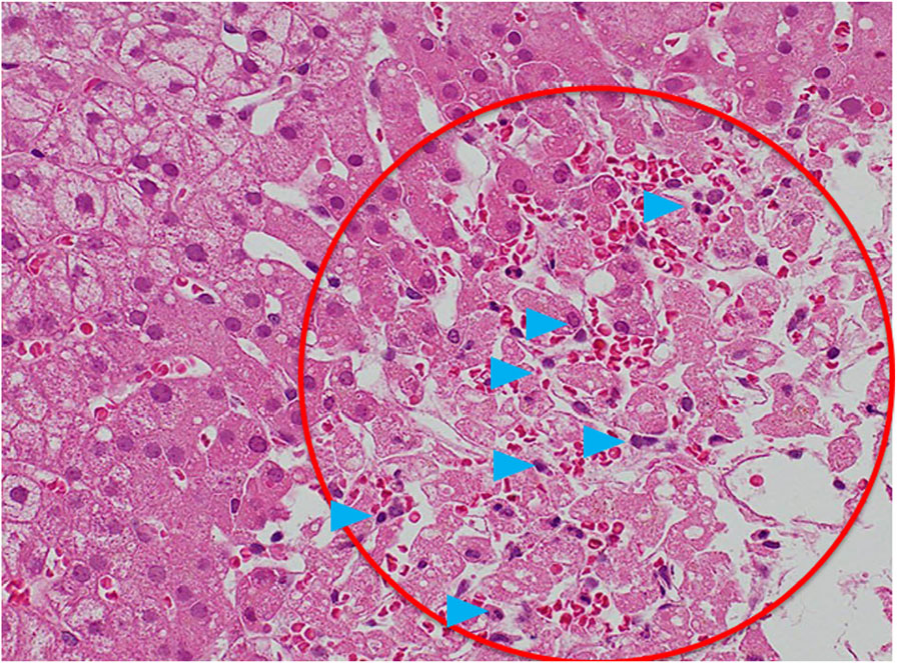
Histopathology findings from the patient’s liver (hematoxylin-eosin; original magnification × 200). Parenchymal necrosis (red circle) and lymphocytic infiltration (blue arrows) were observed

On the second day of hospitalization, we performed a liver biopsy, which showed parenchymal necrosis and lymphocytic infiltration (Fig. 2). On that same night, the patient was hypotensive, with a blood pressure of 80/65 mmHg, and her heart rate was elevated (113 beats/min). Physical examination revealed cyanosis of the lips, distended external jugular veins, pretibial edema in both legs, and coarse crackles over the lower bilateral lung fields.

**Fig. 3:**
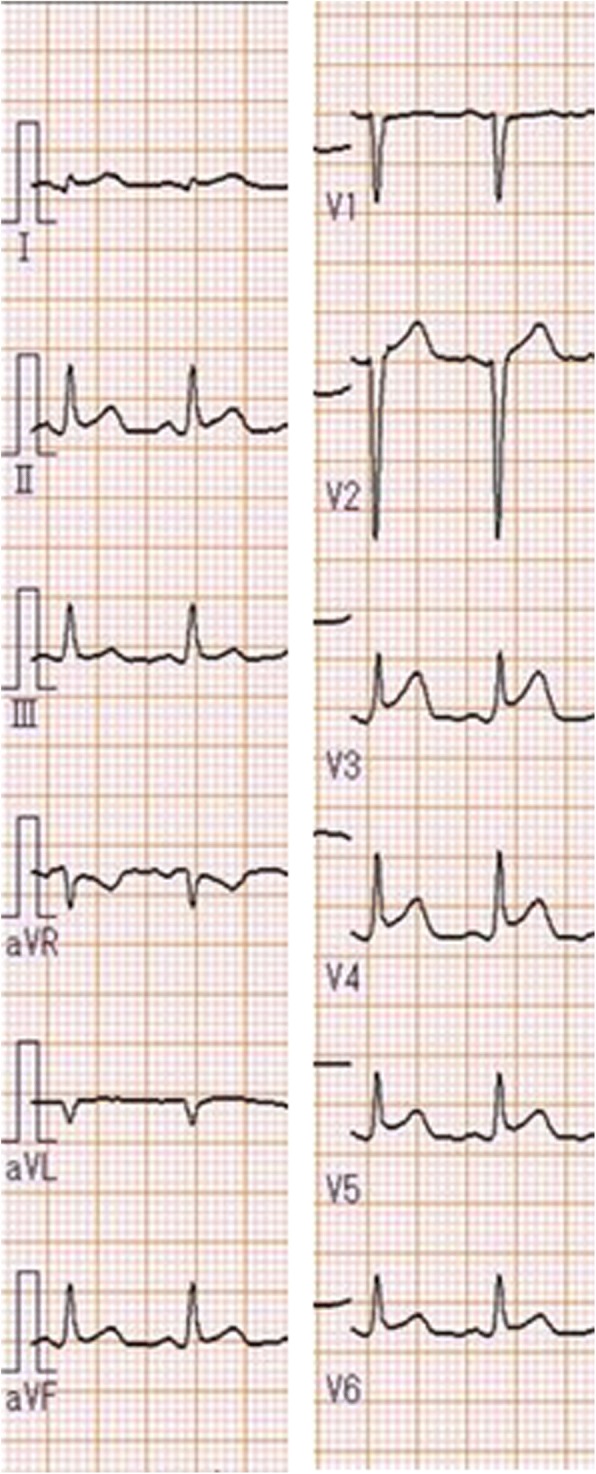
Electrocardiogram finding from the patient. 12-lead electrocardiogram showed diffuse ST-segment elevations, with the exception of V1 and aVR

**Fig. 4:**
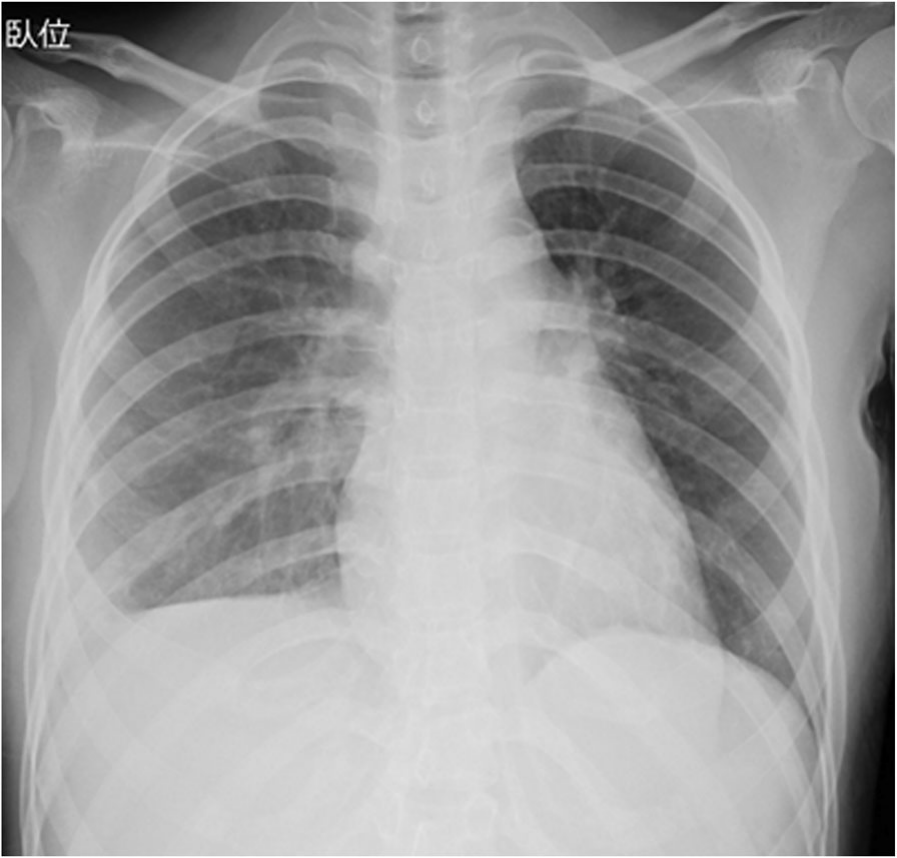
Radiograph findings from the patient’s chest. Chest radiograph showed a normal cardiac size (cardiothoracic ratio, 49%), pulmonary congestion, as well as pleural effusion in the right lung only.

**Fig. 5:**
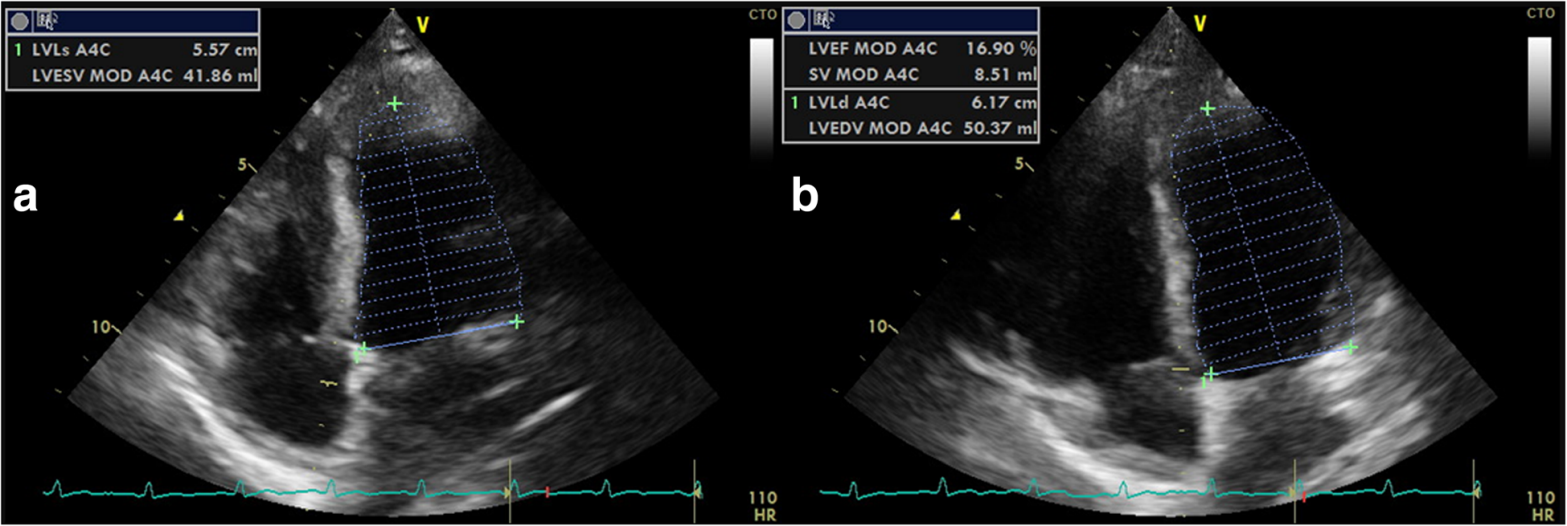
Echocardiographic findings via apical four chamber views showing (**a**) end-systolic and (**b**) end-diastolic volumes. The echocardiography showed a dilated, spherical ventricle with reduced systolic function (left ventricle ejection fraction, 17%) and pericardial effusion

Electrocardiography showed diffuse ST-segment elevation (Fig. 3). Her troponin I, CK level in the blood (CK-MB), and brain natriuretic peptide (BNP) were elevated (troponin T, 0.879 ng/mL [normal value: ≤0.016 ng/mL]; CK-MB, 253 U/L [normal value: ≤5 U/L]; BNP, 1513 pg/mL [normal value: ≤18.4 pg/mL]). Chest radiography showed a normal cardiac size, pulmonary congestion, and pleural effusion in the right lung only (Fig. 4). Echocardiography showed a dilated, spherical ventricle with reduced systolic function (left ventricle ejection fraction [LVEF], 17%) and pericardial effusion (Fig. 5).

**Fig. 6:**
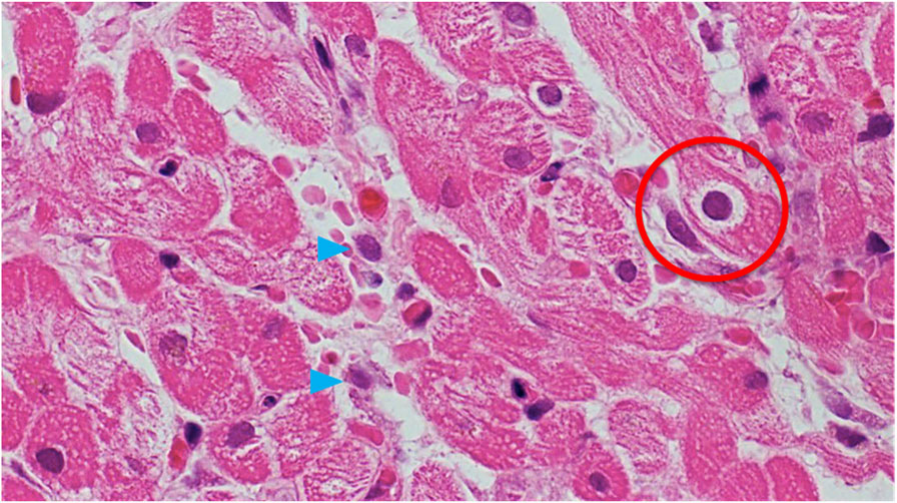
Endomyocardial biopsy (hematoxylin-eosin; original magnification × 400). Lymphocyte infiltration of the myocardium and intranuclear inclusions (red circle) were observed

Cardiac catheterization on day 3 revealed high pulmonary capillary wedge pressure (22 mmHg) and a low cardiac index (2.0 L/min/m^2^). Coronary angiography showed no abnormalities. Endomyocardial biopsy (EMB) was performed via the right internal jugular vein, and five specimens were obtained from the right ventricle side of the interventricular septum. Endomyocardial biopsy findings showed lymphocyte infiltration of the myocardium and intranuclear inclusions, which was confirmed as acute myocarditis (Fig. 6). In response, intravenous administration of dobutamine (4 μg/kg/min) was started on day 3. The patient’s systolic blood pressure increased to approximately 100 mmHg and stabilized, and dobutamine administration was gradually tapered off. On day 10, echocardiography showed normalization of LVEF to 71%, dobutamine was stopped, and laboratory findings were almost normalized as follows: CPK, 143 U/L (standard values: 40–150 U/L); aspartate aminotransferase (AST), 36 U/L (standard values: 11–30 U/L); and alanine aminotransferase (ALT), 278 U/L (standard values: 4–30 U/L). Laboratory findings on day 18 were normalized as follows: CPK, 123 U/L; AST, 18 U/L; ALT, 21 U/L, and BNP, 18 pg/mL. She was eventually discharged on day 19 of her hospital stay.

One serum sample for viral serological testing was collected before the EMBs were obtained. Follow-up serum samples were collected between 2 weeks, 1 month, and 6 months after the initial serum sample. The presence of HSV-specific immunoglobulin M (IgM) was detected upon admission to our hospital, increased at 2 weeks, and returned to normal 6 months later. Additionally, HSV-specific immunoglobulin G (IgG) increased from hospital admission to 2 weeks (Table 2). Other viral serologic tests, including HIV, were negative (Table 3). Based on this finding, we diagnosed the patient with acute myocarditis combined with hepatitis arising from HSV infection.

**Table 2:** Clinical course of herpes simplex virus-specific immunoglobulin M and immunoglobulin G

	Standard value	Admission	2 week	1 month	6 month
HSV-IgM	0.7	11.72	13.27	10.06	0.20
HSV-IgG	1.9	8.0	19.4	20.6	12.0

*HSV* herpes simplex virus, *IgM* immunoglobin M, *IgG* immunoglobin G

**Table 3:** Laboratory data for hepatitis and causative infection agent

T-SPOT	Negative
Antinuclear antibody	40
Anti-M2 Ab	Negative
HBs Ag	Negative
Anti-HBs Ab	Negative
Anti-HBc Ab	Negative
Anti-HCV Ab	Negative
HAV-IgM	0.16
HAV-IgG	0.2
HEV-IgA	Negative
EBVCA-IgM	0.0
EBVCA-IgG	3.3
EBNA-IgG	2.1
HSV-IgM	11.72
HSV-IgG	8.0
CMV-IgM	0.16
CMV-IgG	0.2
PVB19-IgM	Negative
Coxsackievirus	Negative
Adenovirus 3	Negative
Adenovirus 7	Negative
Influenza A/B antigen	Negative
Anti-HIV Ag/Ab	Negative

*Ab* antibody, *CMV* cytomegalovirus, *EBNA* Epstein–Barr nuclear antigen, *EBVCA* Epstein-Barr virus capsid antigen, *HAV* hepatitis A virus, *HBc Ab* hepatitis B core antibody, *HEV* hepatitis E virus, *HBs AG* hepatitis B surface antigen, *HIV* human immunodeficiency virus, *HSV* herpes simplex virus, *IgA, IgG, IgM* immunoglobulin A, G, M; PVB19, parvovirus B19

## Discussion and conclusions

We described a rare case of combined myocarditis and hepatitis caused by HSV infection. To the best of our knowledge, this is the first case of combined myocarditis and hepatitis arising from HSV infection. In our case, both heart and liver biopsies were completed, and lymphocytic infiltration was detected in both biopsies.

Herpes simplex virus hepatitis is an uncommon complication of HSV infection, often leading to acute liver failure (ALF). It is thought to represent less than 1% of all ALF cases, and less than 2% of all viral causes of ALF [9]. Additionally, 24% of HSV hepatitis cases were considered immunocompetent. The remaining patients were either pregnant (23%) or immunocompromised from a previous solid organ, hematopoietic cell transplantation (30%), or immunosuppressive agent (23%) [10]. Symptoms are transient and mild in immunocompetent patients, and serious or fatal in immunocompromised patients [11].

Herpes simplex virus-induced myocarditis is also uncommon. Bowles et al. reported that polymerase chain reactions were positive for HSV in 5 of 624 (0.8%) samples obtained from patients with myocarditis [12]. It has been reported that viral myocarditis may be combined with hepatitis, pancreatitis, nephritis, and encephalitis, but the majority of these reports used postmortem biopsies [6–8]. In our case, both heart and liver biopsies were completed, and lymphocytic infiltration was observed in both biopsies. Moreover, the pathological findings of the EMB specimen did not indicate ischemic hepatitis. We performed a biopsy from multiple organs of the patient, whereas previous reports generally obtained a pathology specimen via necropsy examination. Therefore, reports that obtained a pathology specimen during the patient’s lifetime were rare. Although we could not confirm HSV in our pathological examination, it was serologically apparent that HSV was the cause of the infection.

It is often believed that liver enzyme elevation in a patient with myocarditis stems from ischemic hepatitis. In most cases, we cannot perform liver biopsy because of the patient’s systemic condition and coagulation disorder; however, we must not forget that in a few instances, hepatitis is combined with myocarditis. Acute myocarditis may be combined with hepatitis, pancreatitis, nephritis, and encephalitis; therefore, it is important to determine whether other infectious lesions are present.

In conclusion, we presented a rare case of myocarditis combined with hepatitis that was caused by HSV infection. Acute myocarditis can have concurrence with hepatitis, pancreatitis, nephritis, and encephalitis; thus, determining the presence of other infectious lesions is necessary to provide appropriate treatment for the patient.

## Abbreviations

ALF: Acute liver failure
ALT: Alanine aminotransferase
AST: Aspartate aminotransferase
BNP: Brain natriuretic peptide
CK-MB: CK level in the blood
CPK or CK: Creatine phosphokinase
CV: Coxsackievirus
EMB: Endomyocardial biopsy
HSV: Herpes simplex virus
IgG: Immunoglobulin G
IgM: Immunoglobulin M
LDH: Lactate dehydrogenase
LVEF: Left ventricle ejection fraction
